# Multifocal serous retinopathy with pemigatinib therapy for metastatic colon adenocarcinoma

**DOI:** 10.1186/s40942-021-00305-9

**Published:** 2021-04-23

**Authors:** Oleg Alekseev, Effy Ojuok, Scott Cousins

**Affiliations:** Department of Ophthalmology, Duke University, 2351 Erwin Rd., Durham, NC 27705 USA

**Keywords:** FGFR inhibitor, Pemigatinib, Multifocal serous retinopathy

## Abstract

**Background:**

Pemigatinib is an inhibitor of the fibroblast growth factor receptor (FGFR), recently approved for the treatment of cholangiocarcinoma. FGFR retinopathy is a newly recognized entity, with only two other FGFR inhibitors reported to cause serous retinopathy. Herein, we describe the first published report of a multifocal serous retinopathy secondary to pemigatinib.

**Case presentation:**

A 67-year-old male with stage 4A metastatic colon adenocarcinoma undergoing systemic therapy with pemigatinib was found to have developed bilateral multifocal serous retinopathy. Fundus autofluorescence showed corresponding multifocal hypoautofluorescent foci, whereas fluorescein angiography and indocyanine green angiography were unremarkable. Subretinal fluid resolved rapidly after discontinuation of pemigatinib.

**Conclusions:**

Multifocal serous retinopathy appears to be a class effect of FGFR inhibitors. FGFR retinopathy clinically resembles MEK retinopathy—both feature multifocal subretinal fluid, low visual significance, and quick resolution. However, given that FGFR inhibitors have a broader molecular range than MEK inhibitors, further characterization of FGFR retinopathy is necessary to generate management guidelines.

## Background

Pemigatinib (marketed as Pemazyre) is a small molecule inhibitor of fibroblast growth factor receptor (FGFR) 1, 2, and 3. It was approved by the US Food and Drug Administration in April 2020 for the treatment of locally advanced and metastatic cholangiocarcinoma [1]. It is a potent and selective inhibitor of FGFR tyrosine kinase activity and is effective against tumors that overexpress FGFR. In this report we describe a case of FGFR retinopathy in a 67-year-old man treated with pemigatinib. FGFR retinopathy is a recently recognized entity, with only two FGFR inhibitors reported in the literature to cause serous retinopathy—erdafitinib [2] and AZD4547 [3]. To our knowledge this is the first published report of FGFR retinopathy secondary to pemigatinib.

## Case presentation

A 67-year-old Caucasian male participant in a cancer clinical trial presented for a surveillance ophthalmic examination. His only ocular complaint was mild blurring of vision; his ocular history included a choroidal nevus OD. His medical history was remarkable for stage 4A unresectable colorectal cancer metastatic to the liver, for which he was enrolled in a clinical trial of pemigatinib. The patient had completed 42 days of treatment with 13.5 mg/day of oral pemigatinib at the time of presentation. There were no other changes in his medication regimen during this time period.

**Fig. 1:**
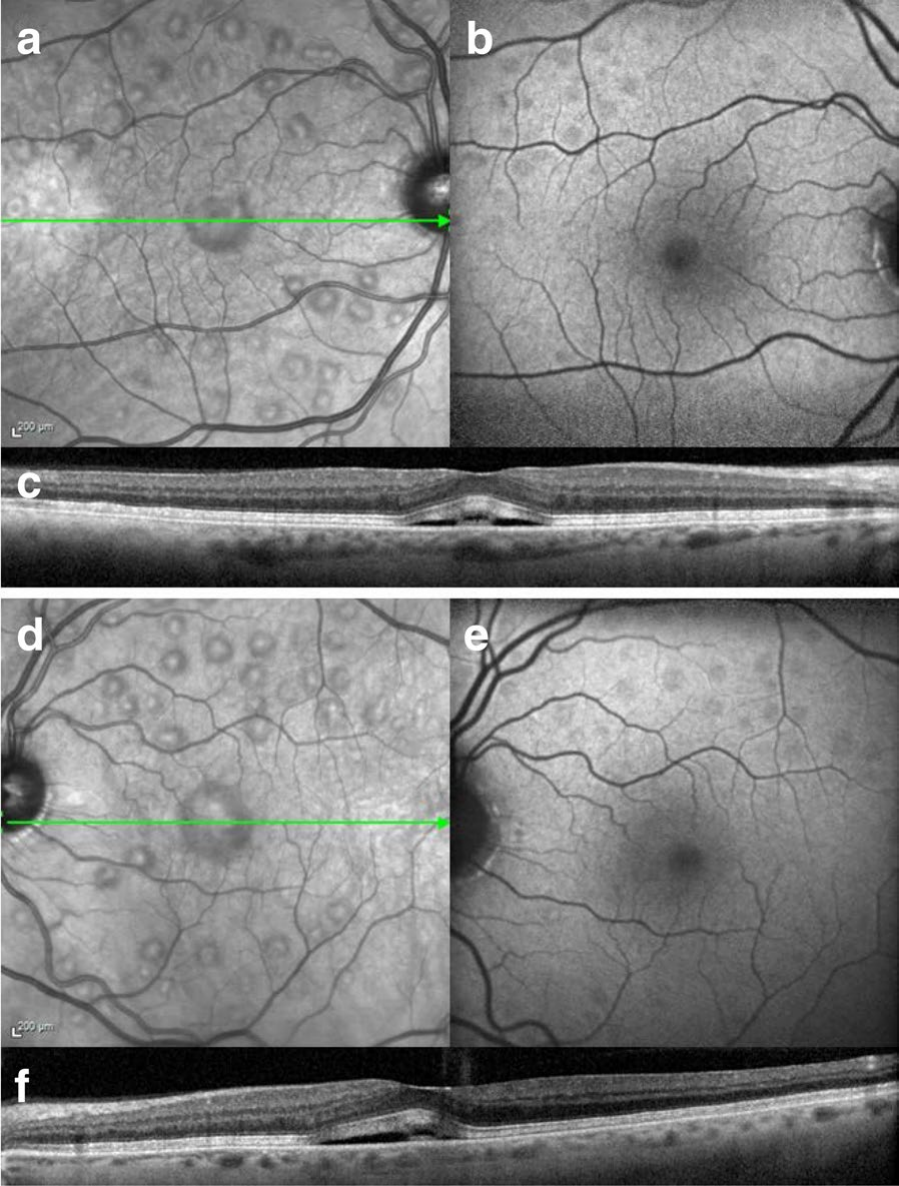
Multimodal imaging of pemigatinib multifocal serous retinopathy. Infrared imaging (**a**, **d**), with the indicated spectral-domain optical coherence tomography sections (**c**, **f**). Multifocal subretinal fluid is present throughout the macula and in the fovea. Fundus autofluorescence imaging (**b**, **e**) shows hypoautofluorescent foci corresponding to areas of subretinal fluid

Ophthalmic examination on presentation demonstrated a best-corrected visual acuity of 20/20-2 in the right eye and 20/20-1 in the left eye. Complete eye examination was remarkable for bilateral multifocal pockets of subretinal fluid (SRF) throughout the macula and subfoveally, which had been absent on a screening examination immediately prior to initiation of pemigatinib. Spectral-domain optical coherence tomography (SD-OCT) showed the presence of central and extrafoveal multifocal SRF, as well as thickening of the interdigitation zone (Fig. 1). In accordance with the clinical trial protocol, pemigatinib treatment was discontinued. Subsequent 5-day re-evaluation showed almost complete resolution of SRF, as well as unremarkable fluorescein angiography (FA) and indocyanine green angiography (ICGA) (except for the known nevus). Notably, fundus autofluorescence (FAF) imaging revealed hypoautofluorescent foci correlating to SRF pockets (Fig. 1).

## Discussion and conclusions

FGFR retinopathy is an emerging entity with limited published information. Retinopathy secondary to another FGFR inhibitor, erdafitinib, was recently reported, though the SRF was found only centrally without the multifocal presentation [2]. A phase II study of the FGFR inhibitor AZD4547 for the treatment of mesothelioma reported the presence of SRF detected by OCT in 12/24 (50%) of patients [3]. The pattern of SRF was described as resembling the phenotype of MEK retinopathy; however, imaging data were not published. In this study, the SRF had a minimal effect on visual acuity and was reversible on treatment discontinuation. The multifocal serous retinopathy observed in our patient undergoing pemigatinib therapy appears strikingly similar to multiple reported cases of MEK retinopathy presenting as multifocal SRF [4–6]. This is consistent with the fact that FGFR acts upstream of the MEK kinase in the FGF-MAPK pathway [7]. However, it is important to note that FGFR inhibition has a much broader molecular range, including activation of the PI3K pathway and regulation of intracellular calcium signaling. This is likely to translate into clinically meaningful differences between the ocular side effect profiles of FGFR inhibitors and MEK inhibitors.

The presence of hypoautofluorescent foci on FAF co-localizing with SRF pockets in the setting of normal FA and ICGA is strongly suggestive of underlying retinal pigment epithelial (RPE) pathology. In fact, profound electrooculogram (EOG) abnormalities and anti-RPE antibodies have been reported in patients with serous SRF in the setting of MEK retinopathy [6], which implicates RPE pump function failure as the likely cause of serous SRF accumulation. While RPE damage in MEK retinopathy appears to be reversible, it is unknown if sustained FGFR inhibition could exert long-term or permanent effects.

The multifocal SRF in MEK retinopathy is typically transient and non-vision-threatening. The case presented here also demonstrated excellent visual acuity on presentation and rapid resolution following treatment discontinuation. However, further studies are necessary to characterize the long-term progression of this side effect with sustained pemigatinib therapy. Systematic monitoring of patients treated with FGFR inhibitors is necessary to determine whether MEK retinopathy and FGFR retinopathy can be considered a single clinical entity, or if they are separate but related conditions.

## Data Availability

Data sharing is not applicable to this article as no datasets were generated or analyzed during the current study.

## Abbreviations

EOG: Electrooculogram
FA: Fluorescein angiography
FAF: Fundus autofluorescence
FGFR: Fibroblast growth factor receptor
ICGA: Indocyanine green angiography
MEK: Mitogen-activate protein kinase kinase
PI3K: Phosphoinositide 3-kinase
RPE: Retinal pigment epithelium
SD-OCT: Spectral-domain optical coherence tomography
SRF: Subretinal fluid
